# Editorial: Exploring new therapeutic frontiers in Glaucoma: from molecular targets to patient outcomes

**DOI:** 10.3389/fphar.2025.1624534

**Published:** 2025-05-19

**Authors:** Silvia Sgambellone, Arianna Carolina Rosa, Ana Raquel Santiago, Ivana Josimovic, Laura Lucarini

**Affiliations:** ^1^ Department of Neuroscience, Psychology, Drug Research and Child Health (NEUROFARBA), Section of Pharmacology, University of Florence, Florence, Italy; ^2^ Department of Scienza e Tecnologia del Farmaco (DSTF), University of Turin, Turin, Italy; ^3^ University of Coimbra, Coimbra Institute for Clinical and Biomedical Research (iCBR), Faculty of Medicine, Coimbra, Portugal; ^4^ University of Coimbra, Center for Innovative Biomedicine and Biotechnology (CiBB), Coimbra, Portugal; ^5^ Clinical Academic Center of Coimbra, Coimbra, Portugal; ^6^ Association for Innovation and Biomedical Research on Light and Image (AIBILI), Coimbra, Portugal; ^7^ University of Coimbra, Immunology Institute, Faculty of Medicine, Coimbra, Portugal; ^8^ Division of Medicinal Chemistry, Amsterdam Institute of Molecular and Life Sciences, Faculty of Science, Vrije Universiteit Amsterdam, Amsterdam, Netherlands

**Keywords:** Glaucoma, intraocular pressure (IOP), neuroprotection, cell regeneration, molecular target

Glaucoma, a leading cause of irreversible blindness worldwide, is a multifaceted challenge for both clinicians and researchers. Traditional treatment strategies have focused primarily on lowering intraocular pressure (IOP), but a deeper understanding of the molecular and cellular mechanisms driving retinal degeneration in glaucoma reveals new therapeutic targets. As research evolves, there is a shift towards therapies that not only control IOP but also protect the retinal structures and regenerate damaged tissue. The studies presented in this Research Topic provide insights into novel therapeutic approaches targeting molecular pathways, ferroptosis, neuroprotection, and disease biomarkers, that hold great promise for improving patient outcomes.

One of the most intriguing molecular agents being investigated is resveratrol, a polyphenolic compound found in plants that has gained attention for its neuroprotective properties. Zhang et al. conducted a systematic review and meta-analysis of preclinical studies, highlighting the potential of resveratrol to protect against retinal damage in glaucoma. The analysis of 30 animal studies showed that resveratrol could improve retinal ganglion cell survival, slow retinal thinning, and visual function. These effects were attributed to the modulation of sirtuin 1 (SIRT1) protein expression, suppression of inflammation, and inhibition of apoptosis. While the results are promising, the authors caution that future clinical trials are needed to confirm these effects in humans and explore the therapeutic potential of resveratrol in the treatment of glaucoma (Zhang et al.).

In parallel to these efforts, ferroptosis, a regulated form of cell death characterized by oxidative stress and iron accumulation, has emerged as a major contributor to retinal degeneration in glaucoma. Hao et al. reviewed the role of ferroptosis in various retinal diseases, including glaucoma. They suggested that inhibition of ferroptosis could prevent retinal ganglion cell death and provide neuroprotection in patients with glaucoma. Their review highlighted the potential of agents such as iron chelators and antioxidants to mitigate ferroptosis-induced damage. Although most ferroptosis-targeting therapies are in the preclinical stages, this approach represents an exciting new frontier in glaucoma treatment, offering an alternative mechanism to prevent retinal damage beyond IOP lowering (Hao et al.).

In addition, genomic and proteomic studies are playing an increasingly important role in identifying novel therapeutic targets for glaucoma. Yuan et al. used a Mendelian randomization approach to identify eight circulating plasma proteins associated with primary open-angle glaucoma (POAG). Among these, ROBO1, FOXO3, and ITIH3 were identified as Tier one therapeutic targets. These proteins are involved in critical processes, such as neuroprotection, extracellular matrix regulation, and oxidative stress—key mechanisms contributing to glaucoma pathogenesis. The identification of these proteins offers a promising avenue for the development of targeted therapies that can go beyond IOP control, aiming to protect the retinal structures and prevent further damage. Furthermore, the potential druggability of these proteins was assessed, with existing compounds such as resveratrol suggested as potential agents for targeting these proteins (Yuan et al.).

Another significant contribution to the understanding of glaucoma pathophysiology is the identification of lactoferrin (Lf) as a potential biomarker of disease severity. Wang et al. reported that plasma Lf levels are significantly elevated in patients with glaucoma when compared with healthy controls. Moreover, elevated plasma Lf levels were strongly correlated with disease severity, as reflected by increased disease grading and reduced retinal nerve fiber layer thickness. Their study suggests that Lf may serve not only as a diagnostic biomarker for glaucoma but also as an indicator of disease progression. This finding could lead to the development of new clinical tools for monitoring glaucoma progression and assessing treatment efficacy (Wang et al.).

Taken together, these studies highlight a transformative shift in glaucoma research and treatment. Rather than focusing solely on lowering IOP researchers are increasingly targeting molecular pathways that contribute to retinal damage, neurodegeneration, and oxidative stress. By identifying novel compounds such as resveratrol, and exploring mechanisms such as ferroptosis and proteomic changes, this body of work represents a comprehensive strategy for treating glaucoma through neuroprotective and regenerative approaches. The identification of lactoferrin as a biomarker also opens new doors for monitoring disease progression, providing an additional level of precision in patient care.

As we move forward, the integration of these new therapeutic targets and biomarkers into clinical practice represents both an opportunity and a challenge. The journey from preclinical discovery to clinical application will require rigorous testing, and collaboration across disciplines. However, the emerging focus on neuroprotection, cell regeneration, and molecular-targeted therapies offers hope for better outcomes for glaucoma patients, potentially not only halting disease progression but also repairing the already established damage.

The future of glaucoma treatment lies in a multifaceted approach, combining IOP management with neuroprotection, cellular regeneration, and molecular targeting. As the pathophysiology of glaucoma proves to be multifactorial and systemic, success will depend on collaborative, cross-disciplinary efforts that bridge molecular science, clinical diagnostics, and patient-centred outcomes.

